# PP2A inhibition overcomes acquired resistance to HER2 targeted therapy

**DOI:** 10.1186/1476-4598-13-157

**Published:** 2014-06-24

**Authors:** Martina SJ McDermott, Brigid C Browne, Neil T Conlon, Neil A O’Brien, Dennis J Slamon, Michael Henry, Paula Meleady, Martin Clynes, Paul Dowling, John Crown, Norma O’Donovan

**Affiliations:** 1 Molecular Therapeutics for Cancer Ireland, National Institute for Cellular Biotechnology, Dublin City University, Glasnevin, Dublin 9, Ireland; 2 Cancer Research Program, The Kinghorn Cancer Centre, Garvan Institute of Medical Research Sydney, Sydney, New South Wales, Australia; 3 Division of Hematology-Oncology, Department of Medicine, David Geffen School of Medicine, University of California at Los Angeles, Los Angeles, California, USA; 4 Department of Biology, National University of Ireland, Maynooth, Maynooth, Co, Kildare, Ireland; 5 Department of Medical Oncology, St Vincent’s University Hospital, Elm Park, Dublin 4, Ireland

**Keywords:** HER2, lapatinib, Resistance, eEF2, PP2A

## Abstract

**Background:**

HER2 targeted therapies including trastuzumab and more recently lapatinib have significantly improved the prognosis for HER2 positive breast cancer patients. However, resistance to these agents is a significant clinical problem. Although several mechanisms have been proposed for resistance to trastuzumab, the mechanisms of lapatinib resistance remain largely unknown. In this study we generated new models of acquired resistance to HER2 targeted therapy and investigated mechanisms of resistance using phospho-proteomic profiling.

**Results:**

Long-term continuous exposure of SKBR3 cells to low dose lapatinib established a cell line, SKBR3-L, which is resistant to both lapatinib and trastuzumab. Phospho-proteomic profiling and immunoblotting revealed significant alterations in phospho-proteins involved in key signaling pathways and molecular events. In particular, phosphorylation of eukaryotic elongation factor 2 (eEF2), which inactivates eEF2, was significantly decreased in SKBR3-L cells compared to the parental SKBR3 cells. SKBR3-L cells exhibited significantly increased activity of protein phosphatase 2A (PP2A), a phosphatase that dephosphorylates eEF2. SKBR3-L cells showed increased sensitivity to PP2A inhibition, with okadaic acid, compared to SKBR3 cells. PP2A inhibition significantly enhanced response to lapatinib in both the SKBR3 and SKBR3-L cells. Furthermore, treatment of SKBR3 parental cells with the PP2A activator, FTY720, decreased sensitivity to lapatinib. The alteration in eEF2 phosphorylation, PP2A activity and sensitivity to okadaic acid were also observed in a second HER2 positive cell line model of acquired lapatinib resistance, HCC1954-L.

**Conclusions:**

Our data suggests that decreased eEF2 phosphorylation, mediated by increased PP2A activity, contributes to resistance to HER2 inhibition and may provide novel targets for therapeutic intervention in HER2 positive breast cancer which is resistant to HER2 targeted therapies.

## Introduction

Overexpression of HER2 occurs in approximately 20-25% of breast cancers resulting in an aggressive tumor phenotype associated with a poor clinical outcome [1]. Following receptor dimerization, activation of HER2 occurs through phosphorylation of tyrosine residues in the kinase domain resulting in the activation of downstream signaling cascades, including P13K/AKT/mTOR and MAPK pathways [2]. Anti-HER2 targeted therapies have been successfully developed, including trastuzumab, a humanized monoclonal antibody targeting the extracellular domain of HER2 [3] and lapatinib, a tyrosine kinase inhibitor that targets the intracellular domain of HER2 and EGFR [4]. Trastuzumab revolutionized the treatment of HER2 positive breast cancer, leading to greater overall response rates and survival compared to chemotherapy alone [5]. Trastuzumab has had the most significant clinical benefit effect in the adjuvant treatment setting, reducing recurrence by approximately 50%. In the metastatic setting, a small but significant proportion (9.5%) of patients achieve a durable complete response following trastuzumab-based therapy [6]. However, many patients do not respond, or respond initially but develop progressive disease within 1-2 years due to the development of resistance. Several potential mechanisms of resistance to trastuzumab have been proposed, including, but not limited to: i) loss of PTEN and/or mutation in P13K/AKT [7,8]; ii) expression of truncated or cleaved HER2 (p95-HER2) [9]; iii) ligand-dependent activation of HER3 [10]; iv) crosstalk with IGF-1R [11,12]; and v) failure to inhibit EGFR signaling [13].

Lapatinib inhibits the growth of trastuzumab-refractory tumors, leading to its approval as a treatment for HER2 positive trastuzumab-refractory metastatic breast cancer, in combination with capecitabine [14]. Addition of lapatinib to capecitabine improved the median overall survival time from 64.7 to 75.0 weeks. However, the majority of patients developed progressive disease and ultimately died from their disease. A synergistic interaction between trastuzumab and lapatinib has been reported *in vitro*[15,16] and the NeoALTTO study reported a significant increase in pathological complete response for patients receiving chemotherapy combined with both trastuzumab and lapatinib (51.3%) compared to chemotherapy with either trastuzumab (29.5%) or lapatinib (24.7%) alone [17].

Several proposed mechanisms of acquired lapatinib resistance have been reported, including increased expression and/or activation of: i) AXL, a MET-related membrane bound receptor tyrosine kinase [18]; ii) myeloid cell factor-1 (MCL-1) [19]; iii) X inhibitor of apoptosis protein (XIAP) [20]; iv) SRC-family kinases [21]; v) RelA, leading to disregulation of NFκβ signaling [22]; and vi) ER signaling [23]. A recent study also reports constitutive activation of mTORC1 as a mechanism of acquired lapatinib resistance, with resistant cells exhibiting enhanced sensitivity to mTOR inhibition [24].

In this study we describe the development and characterization of cell line models of acquired lapatinib resistance. We report significant alterations in the phospho-proteome of lapatinib resistant cells and identify increased PP2A activity as a novel mechanism of resistance to HER2 targeted therapy.

## Results

### Development and characterization of a cell line model of acquired lapatinib resistance

**Figure 1:**
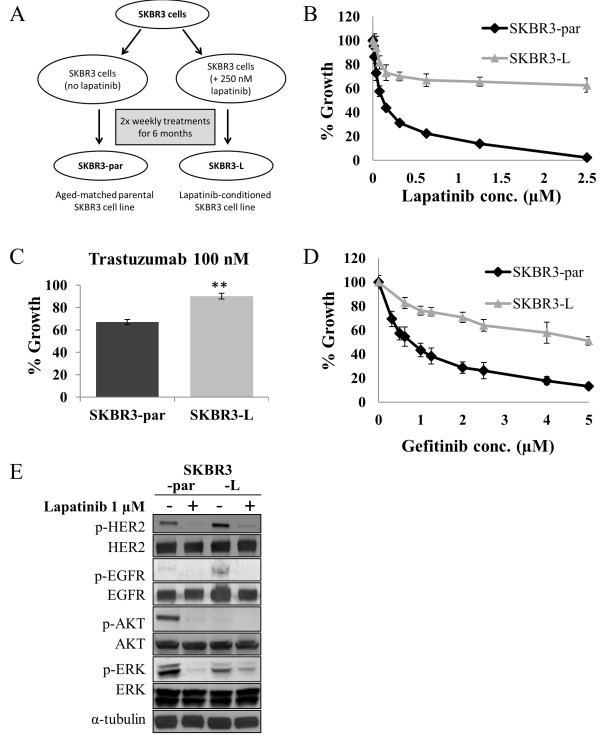
**Development and characterization of a cell-line model of acquired lapatinib resistance. (A)** Schematic depiction of the continuous long-term lapatinib treatment strategy utilized to develop SKBR3-L cells. **(B)** Effect of lapatinib treatment on SKBR3-par and SKBR3-L cells. **(C)** Effect of trastuzumab treatment on SKBR3-par and SKBR3-L cells. **denotes p < 0.01. **(D)** Effect of gefitinib treatment on SKBR3-par and SKBR3-L cells. **(E)** Immunoblot analysis of total and phosphorylated HER2^(Tyr1221/1222)^, EGFR^(Tyr1173)^, AKT^(Ser473)^ and ERK^(Thr202/Tyr204)^ in cells following 24 hr lapatinib treatment. Error bars represent the mean ± SD (n = 3).

We utilized a continuous long-term lapatinib treatment strategy in the HER2-positive breast cancer cell line SKBR3, to develop a cell line model of acquired resistance. The cells were treated twice weekly with 250 nM lapatinib, while parental control cells (SKBR3-par) were cultured alongside the treated cells (Figure 1A). After a period of 6 months continuous treatment, the sensitivity of the treated and un-treated cells was determined. SKBR3-par cells showed similar sensitivity to lapatinib as the SKBR3 cells prior to the 6 month growth period (IC_50_ = 0.1 ± 0.01 μM). In contrast, lapatinib-conditioned cells (SKBR3-L) exhibited a lapatinib IC_50_ of 6.5 ± 0.4 μM, confirming resistance (Figure 1B). SKBR3-L cells were tested for cross-resistance to other HER2 and EGFR targeted agents, trastuzumab and gefitinib. SKBR3-L cells were significantly less sensitive to trastuzumab treatment (p = 0.003) (Figure 1C), and to gefitinib treatment (p = 0.02) (Figure 1D), compared to SKBR3-par cells, suggesting that SKBR3-L cells are resistant to both HER2 and EGFR inhibition. We then examined the expression and phosphorylation of several key members of the HER2 and EGFR signaling pathways in both cell lines. While the expression of HER2, AKT and ERK was unaltered between the two cell lines, SKBR3-L cells exhibited increased expression of EGFR compared to SKBR3-par cells (p = 0.004) (Figure 1E). Significant alterations in each of these proteins were identified when we examined their phosphorylation status. SKBR3-L cells exhibited increased levels of p-HER2 (p = 0.02) and p-EGFR (p = 0.02), and significantly decreased levels of p-ERK (p = 0.01) and p-AKT (p = 0.001). Interestingly, when SKBR3-L cells were treated with lapatinib the levels of p-HER2 and p-EGFR decreased, in a similar manner to SKBR3-par cells (Figure 1E). SKBR3-L cells did not exhibit significant alterations in the expression of XIAP, or SRC phosphorylation. They also did not express ER nor exhibit loss of PTEN compared to SKBR3-par cells (Additional file 1: Figure S1).

### Phosphoproteomic analysis of SKBR3-par and SKBR3-L cells

**Figure 2:**
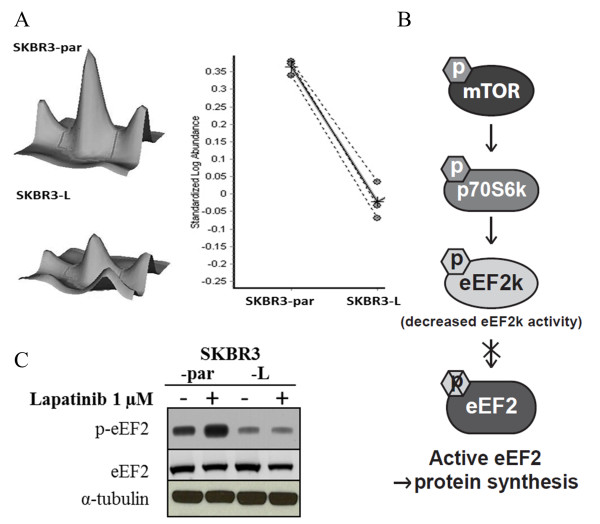
**Phospho-proteomic analysis reveals decreased levels of p-eEF2 in SKBR3-L cells. (A)** Example of a 3D view of p-eEF2 protein abundance with graphs of protein abundance analyzed by DeCyder software in SKBR3-par and SKBR3-L cells. The solid line represents the average of three replicate measurements (dotted lines) of protein abundance. **(B)** Schematic depiction of mTOR-mediated activation of eEF2; active mTOR phosphorylates and activates p70S6k, which in turn phosphorylates and deactivates eEF2k thus preventing the phosphorylation of eEF2 resulting in active eEF2. **(C)** Immunoblot analysis of total and phosphorylated eEF2^(Thr56)^ in SKBR3-par and SKBR3-L cells following 24 hr lapatinib treatment.

The phospho-proteome of SKBR3-par and SKBR3-L cells was compared using a combination of phospho-protein enrichment, 2D-DIGE and mass spectrometry (MS). 2,500 spots were detected and analyzed using DeCyder differential in-gel analysis to produce 3-D images of protein abundance and graphs of relative protein abundance (Figure 2A). Of 2,500 spots detected on the DIGE gels, 81 spots exhibited altered abundance between SKBR3-par and SKBR3-L cells and were picked for identification by MS. When a p-value of ≤ 0.05 and a cut-off of 1.2-fold change were applied, 20 phospho-proteins were significantly higher and 21 were significantly lower in SKBR3-L compared to SKBR3-par cells (Additional file 2: Table S1). The proteins identified were associated with cell growth/differentiation, metabolic processes, transcription, translation, protein folding, immune cell processes and response to stress (Additional file 2: Table S1).

### Validation of decreased eEF2 phosphorylation in SKBR3-L cells

A number of phosphorylated forms of the protein eukaryotic translation elongation factor 2 (eEF2) were significantly differentially regulated in SKBR3-L compared to parental SKBR3 cells; one form was up-regulated, while six forms were down regulated (Additional file 2: Table S1, Figure 2A). eEF2 plays a key role in protein synthesis and lies downstream of the mTOR signaling pathway. The activity of eEF2 is inhibited by its phosphorylation at Thr56 by eukaryotic elongation factor 2 kinase (eEF2k). eEF2k activity is inhibited after phosphorylation at Ser366 by p70S6k, which is in turn phosphorylated and activated by mTOR (Figure 2B). Immunoblotting with a p-eEF2^thr56^ specific antibody confirmed decreased levels of p-eEF2 in SKBR3-L cells compared to SKBR3-par (p = 0.001), while no change in the levels of total eEF2 was observed (Figure 2C). Lapatinib treatment resulted in a significant increase in p-eEF2 in SKBR3-par cells (p = 0.001), but not in SKBR3-L cells, suggesting that an alteration in the signaling pathway upstream of eEF2 may prevent lapatinib-induced phosphorylation of eEF2 and may contribute to lapatinib resistance in SKBR3-L cells.

### mTOR signaling does not mediate decreased phosphorylation of eEF2

**Figure 3:**
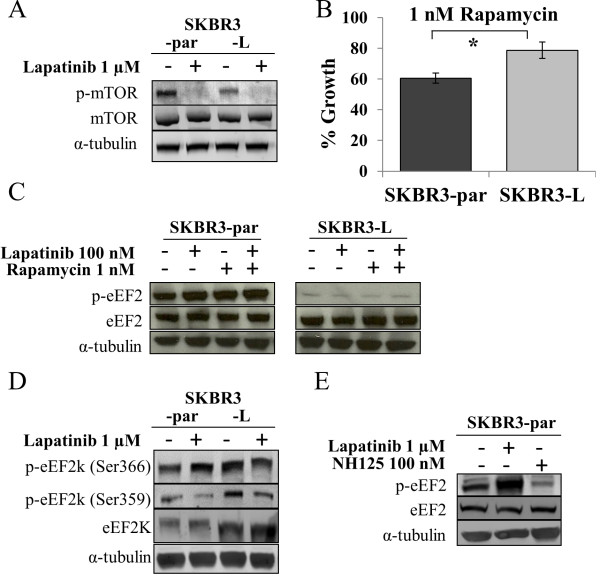
**mTOR and eEF2k mediated regulation of eEF2 phosphorylation. (A)** Immunoblot analysis of total and phosphorylated mTOR^(Ser2448)^ in SKBR3-par and SKBR3-L cells following 24 hr. lapatinib treatment. **(B)** Effect of rapamycin on growth of SKBR3-par and SKBR3-L cells. Error bars represent the mean ± SD (n = 3). **(C)** Immunoblot analysis of total and phosphorylated eEF2^(Thr56)^ following 24 hr. treatment with lapatinib and/or rapamycin. **(D)** Immunoblot analysis of total and phosphorylated eEF2k^(Ser366, 359)^ in SKBR3-par and SKBR3-L cells following 24 hr. lapatinib treatment. **(E)** Immunoblot examining the effect of NH125 alone and in combination with lapatinib on the phosphorylation of eEF2^(Thr56)^ in SKBR3-par cells. *denotes p ≤ 0.05.

To determine if decreased p-eEF2 levels in SKBR3-L cells are mediated by an alteration in mTOR signaling, we examined the levels of p-mTOR^ser2448^ and total mTOR. While the total levels of mTOR were unchanged between the two cell lines, SKBR3-L cells had significantly lower levels of p-mTOR compared to SKBR3-par cells (p = 0.01) (Figure 3A). However, lapatinib treatment significantly decreased the levels of p-mTOR in both SKBR3-par (p = 0.005) and SKBR3-L (p = 0.03) cells. Together, these results suggest that the downregulation of p-eEF2 observed in SKBR3-L cells is not due to increased or constitutive activation of mTOR. In fact, SKBR3-L cells were significantly less sensitive to the mTOR inhibitor, rapamycin, than SKBR3-par cells (p = 0.01) (Figure 3B), suggesting that SKBR3-L cells have reduced dependence on mTOR signaling for growth. Furthermore, SKBR3-par cells treated with rapamycin alone or in combination with lapatinib exhibited a significant increase in p-eEF2 following treatment (Figure 3C). However, treatment of SKBR3-L cells under the same conditions had no effect on p-eEF2. Taken together, these results suggest that the decreased phosphorylation of eEF2 is mediated via a non-mTOR dependent mechanism in SKBR3-L cells.

### eEF2k does not mediate decreased peEF2 in SKBR3-L cells

eEF2 is the sole known substrate of eEF2k, and phosphorylation of eEF2k results in its inactivation, rendering it incapable of phosphorylating eEF2 (Figure 2B). Therefore, we hypothesized that increased p-eEF2k in SKBR3-L cells may account for the decreased levels of p-eEF2 in those cells. eEF2k is phosphorylated in an mTOR-dependent manner at Ser366. Immunoblotting revealed significantly higher levels of both p-eEF2k^ser366^ and total eEF2k in SKBR3-L cells compared to SKBR3-par cells (p-eEF2k, p = 0.04; eEF2k, p = 0.01) (Figure 3D). However, lapatinib treatment of SKBR3-par cells, which increased p-eEF2 levels, also increased p-eEF2k^ser366^ levels (p = 0.03), suggesting that the increase in p-eEF2 levels is not mediated by decreased mTOR-dependent phosphorylation of eEF2k. eEF2k activity can also be inhibited in an mTOR-independent manner through phosphorylation at Ser359 by cyclin dependent kinase 1 (CDK1) or mitogen-activated protein kinase 13 (MAPK13). SKBR3-L cells have significantly higher levels of p-eEF2k^ser359^ compared to SKBR3-par cells (p = 0.03), and SKBR3-par cells exhibited decreased p-eEF2k^ser359^ following lapatinib treatment (p = 0.04). However, SKBR3-L cells also exhibited a similar decrease in p-eEF2k^ser359^ following lapatinib treatment (p = 0.06), despite exhibiting no alteration in p-eEF2 in response to lapatinib, thus suggesting that the decreased levels of p-eEF2 in SKBR3-L cells is not mediated by altered expression and/or phosphorylation of eEF2k.

We also examined inhibition of eEF2k activity in SKBR3-par cells to determine if decreased eEF2k activity would cause decreased p-eEF2 levels and reduced sensitivity to lapatinib. Treatment of SKBR3-par cells with the eEF2k inhibitor NH125 resulted in a significant decrease in p-eEF2 (p = 0.01) (Figure 3E). Treatment of SKBR3-par cells with NH125 in combination with lapatinib did not result in a significant decrease in lapatinib sensitivity compared to lapatinib alone (Additional file 3: Figure S2). This suggests that inhibition of eEF2k activity alone is not sufficient to cause the level of lapatinib resistance observed in SKBR3-L cells.

### Dephosphorylation of eEF2 by PP2A

**Figure 4:**
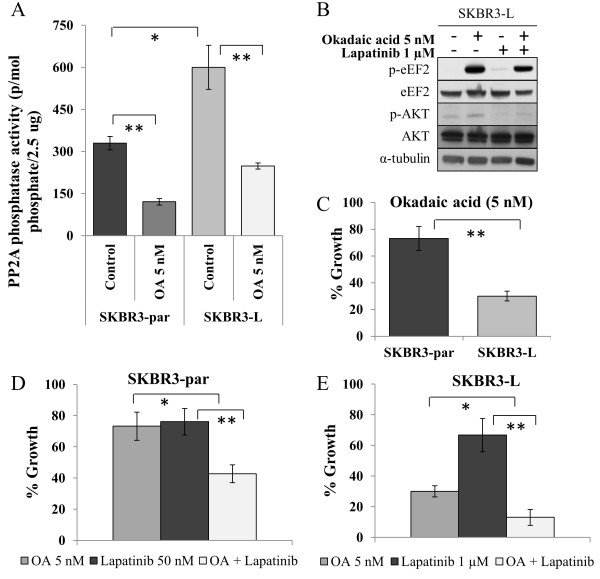
**The role of PP2A in eEF2 phosphorylation. (A)** Activity of PP2A in SKBR3-par and SKBR3-L cells, untreated and treated with 5 nM OA for 24 hr. **(B)** Immunoblot examining the effect of OA alone and in combination with lapatinib on levels of total and phosphorylated eEF2^(Thr56)^ and AKT^(Ser473)^. **(C)** Effect of 5 nM okadaic acid (OA) on growth of SKBR3-par and SKBR3-L cells. **(D)** Effect of OA alone and in combination with lapatinib on the growth of SKBR3-par cells and **(E)** SKBR3-L cells. Error bars represent the mean ± SD (n = 3). *denotes p ≤ 0.05, **denotes p ≤ 0.01.

Protein phosphatase 2A (PP2A) activity results in the direct dephosphorylation of multiple proteins including eEF2 and AKT. Therefore we hypothesized that increased PP2A activity in SKBR3-L cells may account for the decreased levels of p-eEF2 and p-AKT observed. PP2A activity was measured in extracts from SKBR3-par and SKBR3-L cells using a phosphatase assay under conditions selective for PP2A activity. SKBR3-L cells displayed 1.8-fold higher PP2A activity compared to SKBR3-par cells (p = 0.02) (Figure 4A). Okadaic acid (OA), a potent lab-grade PP2A inhibitor was used to confirm PP2A activity, and caused a decrease in PP2A activity in both cells lines. Treatment of SKBR3-L cells with OA resulted in a significant increase in the levels of p-eEF2 (p = 0.02) and this effect was sustained when OA was combined with lapatinib (p = 0.03) (Figure 4B). Levels of p-AKT were also increased following OA treatment, although to a lesser extent. SKBR3-L cells were significantly more sensitive to OA (5 nM) treatment than SKBR3-par cells in proliferation assays (69.9 ± 3.7% versus 26.8 ± 9.1% growth inhibition, p = 0.007) (Figure 4C). Combined treatment of SKBR3-par cells with lapatinib and OA resulted in significantly greater inhibition of growth compared to either lapatinib (p = 0.007) or OA alone (p = 0.012) (Figure 4D). Importantly, significantly enhanced growth inhibition was also observed in SKBR3-L cells following combined treatment compared to single agent lapatinib (p = 0.005) or OA (p = 0.012) (Figure 4E). In contrast, treatment of SKBR3-L cells with trastuzumab and okadaic acid did not result in enhanced growth inhibition compared to okadaic acid alone (Additional file 4: Figure S3A). To determine if okadaic acid combined with lapatinib was cytostatic or cytocidal, cell cycle assays were performed. Lapatinib alone (500 nM) induced G1 arrest (51.8 ± 1.6% compared to 41.8 ± 2.9% for untreated cells), OA alone induced a small increase in the sub-G1 population (12.8 ± 6.3% versus 8.9 ± 3.4% for untreated cells) and the combination induced an increase in the sub-G1 fraction (23.1 ± 8.5%) compared to either lapatinib (9.1 ± 6.7%, p = 0.044) or OA (12.8 ± 6.3%, p = 0.083) alone (Additional file 4: Figure S3B).

To determine if PP2A activation plays a causative role in the development of acquired lapatinib resistance, SKBR3 cells were treated with the PP2A activator, FTY720. Following a 24 hour treatment with FTY720 (2.5 μM), lapatinib sensitivity was decreased in the SKBR3 parental cells, with a 5.3-fold increase in the lapatinib IC_50_ (133.5 ± 2.3 nM compared to 25.9 ± 3.0 nM) (Additional file 5: Figure S4). FTY720 alone, at 2.5 μM, did not significantly affect the growth of the SKBR3 parental cells (2.5 ± 0.2% growth inhibition).

### PP2A dephosphorylation of eEF2 in HCC1954-L cells

**Figure 5:**
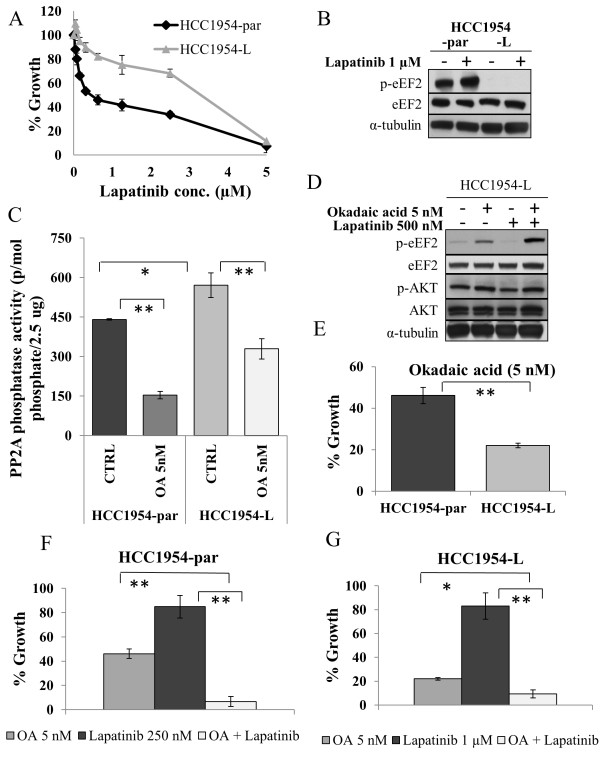
**PP2A regulates eEF2 phosphorylation in an additional cell line model of acquired lapatinib resistance. (A)** Effect of lapatinib on growth of HCC1954-par and HCC1954-L cells. **(B)** Immunoblot analysis of total and phosphorylated eEF2^(Thr56)^ in HCC1954-par and HCC1954-L cells following 24 hr lapatinib treatment. **(C)** Activity of PP2A in HCC1954-par and HCC1954-L cells, untreated and treated with 5 nM OA for 24 hr. **(D)** Immunoblot examining the effect of okadaic acid (OA) alone and in combination with lapatinib on levels of total and phosphorylated eEF2^(Thr56)^ and AKT^(Ser473)^. **(E)** Effect of 5 nM OA on growth of SKBR3-par and SKBR3-L cells. **(F)** Effect of OA alone and in combination with lapatinib on the growth of HCC1954-par cells and **(G)** HCC1954-L cells. *denotes p ≤ 0.05, **denotes p ≤ 0.01. Error bars represent the mean ± SD (n = 3).

To validate the potential of altered PP2A activity as a mechanism of acquired lapatinib resistance, we developed a second cell line model of acquired lapatinib resistance (HCC1954-L) using a similar long-term lapatinib treatment strategy. Continuous treatment of HCC1954 cells resulted in the development of HCC1954-par cells and HCC1954-L cells with lapatinib IC_50_ values of 0.42 ± 0.02 μM and 2.67 ± 0.08 μM, respectively (Figure 5A). HCC1954-L cells have significantly lower levels of p-eEF2 compared to HCC1954-par cells (p = 0.002), and lapatinib treatment significantly increased p-eEF2 levels in HCC1954-par cells, with no effect on the levels of p-eEF2 in HCC1954-L cells (Figure 5B). HCC1954-L cells had 1.3-fold higher PP2A activity compared to HCC1954-par cells (p = 0.04) (Figure 5C). OA treatment of HCC1954-L cells increased p-eEF2 and p-AKT levels (Figure 5D). HCC1954-L cells exhibited significantly greater growth inhibition in response to OA treatment (78.0 ± 1.1%) compared to HCC1954-par cells (53.9 ± 3.9%) (p = 0.005) (Figure 5E). Combined treatment with lapatinib and OA was significantly more effective than either agent alone, in both HCC1954-par (Figure 5F) and HCC1954-L cells (Figure 5G).

## Discussion

Treatment of HER2-positve breast cancer patients is hindered by resistance to HER2-targeted therapies. In the present study, we have described the development and characterization of cell line models of acquired lapatinib resistance and identified a novel potential target for the treatment of HER2-positive breast cancer that does not respond to anti-HER2 therapies.

The SKBR3-L cell line model of acquired lapatinib resistance was developed by continuous long-term (6 month) exposure to low dose (250 nM) lapatinib, in contrast to previous studies which utilized high-dose lapatinib and/or dose escalation procedures [18-24]. The median peak plasma concentration of lapatinib reported in patients receiving 1200 mg lapatinib (once daily) was 1.2 μg/ml (2.1 μM) and the median steady-state trough concentration was 0.3 μg/ml (0.5 μM), with a range of 0.2-0.5 μg/ml [25]. To our knowledge, the models of acquired lapatinib resistance presented here, SKBR3-L and HCC1954-L, are the first to show that extended exposure to concentrations of lapatinib in the steady state trough concentration range, results in significant lapatinib resistance, with resulting lapatinib IC_50_ values significantly higher than the concentration used for conditioning. Interestingly, SKBR3-L cells do not exhibit any significant alterations in XIAP, SRC or ER expression, as have been described in other models of lapatinib resistance [20,21,23]. Furthermore, Jegg et al [24] recently developed another SKBR3 model of acquired lapatinib resistance using escalating doses of lapatinib, which resulted in constitutive activation of mTOR; we did not observe this alteration in the SKBR3-L cells. Therefore, the resistance mechanisms triggered by lapatinib exposure may be concentration dependent.

SKBR3-L cells exhibit cross-resistance to both trastuzumab (anti-HER2) and gefitinib (anti-EGFR). Cross-resistance to trastuzumab has previously been reported in a cell line model of acquired lapatinib resistance [26], but to our knowledge cross-resistance to gefitinib has not previously been reported. Interestingly, acquired trastuzumab resistance may not be accompanied by cross-resistance to anti-HER2 tyrosine kinase therapy, as trastuzumab conditioned breast cancer cell lines retained lapatinib sensitivity [15]. Phosphorylation of both HER2 and EGFR in SKBR3-L cells is inhibited by lapatinib treatment, which is consistent with other cell line models of acquired lapatinib resistance [21,22,26], suggesting that the mechanisms of resistance to lapatinib, trastuzumab and gefitinib lie downstream of HER2/EGFR.

PI3K mutation and/or loss of PTEN have been shown to contribute to maintained activation of AKT and are associated with innate resistance to trastuzumab [8] and lapatinib [27]. In our study, SKBR3-L cells exhibit a dramatic decrease in p-AKT compared to parental cells, suggesting a decreased dependence on the P13K pathway for growth and survival, and no alteration in PTEN levels was observed. A decrease in p-MAPK was also observed indicating that numerous alterations in protein phosphorylation/kinase signaling pathways are occurring simultaneously in SKBR3-L cells. Thus, we compared the global phosphorylation pattern of SKBR3-L and SKBR3-par cells using 2D-gel electrophoresis coupled with MALDI-Tof/Tof mass spectrometry analysis, a technique which has been successfully applied to the study of altered protein phosphorylation [28]. eEF2 showed the largest decrease in phosphorylation levels in SKBR3-L compared to the SKBR3 parental cells and was selected for further analysis.

eEF2 plays a critical role in regulating protein synthesis. Phosphorylation of eEF2^thr56^ prevents it from binding to the ribosome, which results in the termination of protein translation [29]. eEF2 is phosphorylated by eEF2k [30], the activity of which is negatively regulated by phosphorylation at Ser366 via mTOR signaling [31] and by phosphorylation at Ser359 by MAPK13 or CDK1 [32], preventing its phosphorylation of eEF2. SKBR3-par cells responded to lapatinib treatment with increased p-eEF2^thr56^; however, no increase in p-eEF2 was observed in SKBR3-L cells, suggesting inhibition of protein synthesis in SKBR3-par cells only. Consistent with this we found higher levels of phosphorylated eEF2k at both Ser366 and Ser359, and increased expression of eEF2k, suggesting that decreased eEF2k activity may contribute to decreased eEF2 phosphorylation in SKBR3-L cells. NH125 is an eEF2k inhibitor which decreases the phosphorylation of eEF2 [33]. NH125 treatment decreased eEF2k activity in SKBR3-par cells and significantly decreased eEF2 phosphorylation. However, the effect of eEF2k inhibition on lapatinib sensitivity was limited, suggesting that decreased eEF2k activity is not a major mediator of acquired lapatinib resistance. We also examined if inhibition of mTOR signaling by rapamycin alters the phosphorylation of eEF2 in SKBR3-L cells. We found that SKBR3-L cells have lower p-mTOR levels, are less sensitive to rapamycin and do not exhibit increased eEF2 phosphorylation in response to rapamycin, suggesting that constitutive activation of mTOR is not a major regulator of eEF2 phosphorylation in this lapatinib-resistant model. Although eEF2k is the only known kinase to phosphorylate eEF2^thr56^, a recent study reports a novel mode of eEF2 regulation, whereby phosphorylation of eEF2 at Ser595 by CDK2 stimulates phosphorylation at Thr56 by eEF2k [34]; further investigation is required to determine if Ser595 phosphorylation is altered in SKBR3-L cells, however our studies with NH125 suggest that eEF2k activity does not play a significant role in acquired lapatinib resistance in SKBR3-L cells.

Phosphorylation of eEF2 is also negatively regulated by PP2A, a family of protein phosphatases that functions to dephosphorylate multiple proteins [35]. Transgenic mice overexpressing PP2A exhibit increased eEF2 dephosphorylation [36], and inhibitors of PP2A attenuate eEF2 dephosphorylation [37]. While PP2A was initially reported to be a tumor suppressor [38], recent studies have found that only some individual subunits of PP2A have tumor suppressor functions that are context dependent; PP2A activity has been associated with leukemic cell survival [39], pancreatic cancer cell growth [40], and has been correlated with poor survival in glioblastoma [41]. While altered PP2A activity has not previously been reported as a mechanism of lapatinib resistance, there is evidence that inhibition of HER2 signaling can result in increased PP2A activity in a lapatinib sensitive breast cancer cell line [42]. However, the same study also reported a correlation between decreased PP2A activity and tumor progression for HER2 positive breast tumors, which is more consistent with the role of PP2A as a tumor suppressor. We examined the activity of PP2A in SKBR3-L and HCC1954-L cells and found significantly higher activity of PP2A and increased sensitivity to the PP2A inhibitor OA, both alone and in combination with lapatinib, compared to parental cells. The combined treatment with OA and lapatinib resulted in an increase in the percentage of cells in sub-G1, in a cell cycle assay, which suggests an increase in apoptosis induction. Furthermore, inhibition of PP2A restored the phosphorylation of eEF2 in both resistant cell lines. These results suggest that increased PP2A activity contributes to the acquired resistant phenotype. Pre-treatment of SKBR3 parental cells with the PP2A activator FTY720 [43] decreased sensitivity to lapatinib, suggesting a causative role for PP2A activation in the development of acquired lapatinib resistance in this model. PP2A inhibition did not appear to improve response to trastuzumab in the SKBR3-L cells, suggesting that there may be other alterations in the cells that contribute to trastuzumab resistance. Further work is required to elucidate the mechanism leading to increased PP2A activity in acquired anti-HER2 therapy resistance and the role of PP2A as a potential mediator of acquired resistance. Assessment of PP2A levels and/or activity in tumor samples following exposure to lapatinib will be required to determine the clinical significance of this potential resistance mechanism.

Fostriecin, a potent and selective inhibitor of PP2A, was tested as a cancer therapy in a phase I clinical trial but the trial was suspended due to drug instability [44]. Cantharidin, an inhibitor of PP1, PP2A, PP4 and PP5, has been used as an anticancer agent in traditional medicine but has been largely ignored by Western medicine due to toxicities. However, less toxic and more selective derivatives of cantharidin have been developed and have been shown to enhance response to chemotherapy in preclinical models of glioma, sarcoma and metastatic pheochromocytoma [45-48]. A phase I trial of LB-1, alone and in combination with docetaxel, in advanced solid tumors is currently ongoing (NCT01837667).

## Conclusions

In summary, we report increased PP2A activity and activated eEF2 in two cell lines models of acquired lapatinib resistance. We demonstrate that PP2A inhibition significantly enhances the growth inhibitory effects of lapatinib in both sensitive and resistant cell lines. These data provide novel potential biomarkers of lapatinib resistance and support the rationale for further investigation of combined PP2A and HER2 inhibition as a therapeutic strategy for refractory HER2 positive breast cancer.

## Methods

### Cells lines and reagents

SKBR3 and HCC1954 cells were obtained from ATCC and maintained in RPMI 1640 (Sigma-Aldrich) with 10% fetal calf serum (GE Healthcare). Stock solutions (10 mM) of lapatinib (provided by GlaxoSmithKline), gefitinib (Sequoia Research Products), Okadaic acid (SantaCruzBiotechnology), NH125 (Calbiochem) and FTY720 (Sigma-Aldrich) were prepared in dimethyl sulfoxide. Trastuzumab was obtained from St. Vincent’s University Hospital, Dublin Ireland.

### Proliferation assay

Proliferation was measured using an acid phosphatase assay; 1.5-3 × 10^3^ cells were seeded in 96-well plates, incubated overnight prior to addition of drug. After 5 days cells were washed with PBS. 10 mM paranitrophenol phosphate substrate (Sigma-Aldrich) in 0.1 M sodium acetate buffer with 0.1% Triton X (Sigma Aldrich) was added to each well and incubated at 37°C for 2 hours. 50 μl of 1 M NaOH was added and the absorbance was read at 405 nM (reference—620 nM). Growth of drug treated cells was calculated relative to control untreated cells in biological triplicate.

### Establishment of lapatinib-resistant cell lines

The IC_70_ concentration of 4 day lapatinib treatment on SKBR3 cells was determined using the acid phosphatase method. SKBR3 cells were treated with 250 nM lapatinib twice weekly for 6 months, the IC_50_ of lapatinib in the parental and conditioned cells was measured to determine resistance, and the resulting cell line was denoted as SKBR3-L. Parental cells (SKBR3-par cells) were cultured alongside the conditioned cells. HCC1954-L and HCC1954-par cells were also established using the above technique using 1 μM lapatinib; the IC_70_ concentration for HCC1954 cells treated with lapatinib for 4 days. Resistant cells were maintained continuously in the presence of lapatinib, however, prior to all assays lapatinib was removed from the cells for 1 week.

### Cell cycle assay

2.5 × 10^4^ cells were seeded per well in a 24 well plate and incubated at 37°C. After 24 hours, 3 nM okadaic acid, 100 nM or 500 nM lapatinib, or a combination of 3 nM okadaic acid with 100 nM or 500 nM lapatinib were added to each well. Following 48 hours incubation, media was collected and the wells were rinsed with PBS. Cells were trypsinized and added to collected media. The media was centrifuged at 300 × g for 5 minutes and the supernatant was aspirated. 150 μl of PBS was added to the pellet, re-suspended and transferred to a round bottom 96-well plate. The plate was centrifuged at 300 × g for 5 minutes and the supernatant was removed, leaving approximately 15 μl per well. The remaining volume was resuspended and 200 μl of ice cold 70% ethanol was added. The plate was stored for 2 hours at -20°C. After fixing the cells, the plate was spun at 450 × g for 5 minute. The supernatant was removed, 200 μl of PBS was added and the plate was centrifuged again at 450 × g for 5 minutes. The PBS was removed and 200 μl of Guava Cell Cycle reagent was added to each well. The cells were resuspended and stored at room temperature in the dark for 30 minutes. Cells were then analyzed on the Guava EasyCyte (Guava Technologies) and the data was analyzed using Modfit LT software (Verify Software House).

### 2D-gel electrophoresis and MALDI-TOF mass spectrometry

SKBR3-par and SKBR3-L cells were cultured to 90% confluent in biological triplicate, total protein was extracted and phosphoprotein fragments were concentrated using the Pierce™ Phosphoprotein Enrichment Kit (Pierce Biotechnology) according to the kit instructions using the lysis buffer provided supplemented with CHAPS (0.25%), 1X Halt Protease Inhibitor EDTA-free and 1X Halt Phosphatase Inhibitor Cocktail (Pierce Biotechnology). Cy-labelled samples were run on 2D-DIGE gels as previously described [28]. To account for any labeling bias by the different dyes, reciprocal labeling experiments were carried out. Any proteins showing differential effects specific to the use of the Cy3/Cy5 dyes were removed from the analysis. A mixed (equal pool of all samples) internal standard labeled with Cy2 was included. This internal standard, which contains every protein present across all samples in the experiment, was applied to all the replicate gels together with distinct Cy3- and Cy5-labelled samples, thereby ensuring inter- and intra-gel matching. The unique signal of every protein in the internal standard was used for quantitative comparisons within each gel and for normalization of the spots across the various gels. Gels were scanned with the Typhoon 9400 Variable Mode Imager (GE Healthcare). The biological variation analysis (BVA) module of Decyder 6.5 was used to generate lists of differentially expressed proteins. Preparative gels stained with colloidal CBB stain (Sigma-Aldrich) were scanned using the Typhoon and images were then matched to the Master gel image generated from the DIGE experiment. Spots of interest were selected and tryptic digestions were performed with the Ettan Digestor robot (GE Healthcare), using 2.5 ng/ml trypsin in 40 mM NH_4_HCO_3_ in 10% acetonitrile solution overnight at 37°C. Digested peptides were eluted in 70% acetonitrile, lyophilized and resuspended in 0.1% TFA solution in 50% acetonitrile. The peptide extract added to a 384 spot MALDI sample plate (Applied Biosystems) and supplemented with 0.5 μl of a 5 mg/ml solution of recrystalized α-cyano-4-hydroxy-trans-cinnamic acid matrix plus 10 mM NH_4_H_2_PO_4_ in 50% acetonitrile/water containing 0.1% TFA. MALDI mass spectra were generated using a 4800 TOF/TOF Proteomics Analyzer instrument (Applied Biosystems), using a Pep4 internal calibrant. The MS and MS/MS data were combined and searched against a number of databases using GPS Explorer software (Applied Biosystems) and a local MASCOT (Matrix Science, London, UK) search engine for protein identification. Pathways associated with the phospho-proteins identified were determined using Panther software ((http://www.pantherdb.org/) [49,50].

### Immunoblot analysis

30 μg of protein were separated on polyacrylamide gels (Lonza), transferred to nitrocellulose membranes (Invitrogen) and blocked for 1 hour in blocking solution (2.5% milk powder (Biorad) in PBS containing 0.1% Tween-20 (PBS-T)). Membranes were incubated at 4°C overnight with primary antibody at a 1:1000 dilution in blocking solution (unless otherwise stated) against p-HER2^tyr1221/1222^, p-EGFR^tyr1173^, AKT, p-AKT^ser473^, ERK, pERK^thr202/tyr204^, eEF2, p-eEF2^thr56^, mTOR, p-mTOR^ser2448^, eEF2k, p-eEF2k^ser366^ (Cell Signaling Technology), p-eEF2k^ser359^ (1:200) (SantaCruzBiotechnology), HER2 (Calbiochem), EGFR (1:250) (Neomarkers), and α-tubulin, anti-mouse and anti-rabbit secondary antibodies (Sigma-Aldrich). Detection was performed using Luminol (SantaCruzBiotechnology) or ECL Advance (GE Healthcare).

### PP2A activity assay

PP2A activity was measured using a serine/threonine phosphatase assay kit (Promega). Briefly, cells were lysed (50 mM Tris-HCl pH 7.5, 0.05% Triton X-100, 0.1 mM EDTA, 0.5 mM PMSF, 0.05% β-mercaptoethanol, 10% glycerol) with protease inhibitors (Sigma-Aldrich) for 40 mins with frequent vortexing. Free phosphate was removed by filtering through Sephadex G25 column and protein concentration was determined using the Bradford assay (Biorad). 2.5 μg of sample was loaded to a 96-well plate with peptide substrate RRA(pT)VA in PP2A-specific reaction buffer (250 mM imidazole pH 7.2, 1 mM EGTA, 0.1% β-mercaptoethanol, 0.5 mg/ml BSA), at 37°C for 20 min. After incubation 50 μl of molybdate dye/additive mixture was added and allowed to develop for 20 mins, the plate was read at 630 nM.

### Statistical analysis

Each experiment was performed with a minimum of three biological replicates for each condition. IC_50_ concentrations were determined using CalcuSyn software (Biosoft). Densitometry was performed using ImageQuant software (GE Healthcare) whereby phosphoprotein was normalized to total protein and the results were normalized to α-tubulin as a loading control. Analysis of the difference of comparisons in protein levels and response to treatment was performed using the Student t-test (two-tailed with unequal variance).

## Competing interests

JC and NOD received research funding from GSK, and JC and DJS received speaker’s honoraria from GSK.

## Authors’ contributions

MMcD developed HCC1954-L cells, performed proliferation, immunoblotting and PP2A activity assays and drafted the manuscript. BB developed SKBR3-L cells. BB, MH and PD performed phosphoproteomic profiling. NC performed the cell cycle assays and the FTY720 treatment assays. PD, MC and PM contributed to the study design and data analysis for the phosphoproteomic profiling. NOB, BB and NOD helped to draft the manuscript. NOD conceived of and designed the study. JC and DS contributed to design and coordination of the study. All authors read and approved the final manuscript.

## Supplementary Material

Additional file 1: Figure S1 Analysis of previously published lapatinib resistance mechanisms. Immunoblot analysis of XIAP, estrogen receptor α (ER), total and phosphorylated SRC and PTEN in SKBR3-par and SKBR3-L cells following 24 hr. lapatinib treatment. Click here for file

Additional file 2: Table S1 Phosphoproteins in SKBR3-L compared to SKBR3-par cells. List of identified phosphoproteins with ≥ 1.2-fold increase or decrease in levels in SKBR3-L compared to SKBR3 cells. Click here for file

Additional file 3: Figure S2 Concentration dependent effect on NH125 on lapatinib sensitivity. SKBR3-par cells were pre-treated with either 100 nM (A, B and C) or 250 nM (D, E and F) NH125 for 24 hours, after which time NH125 was removed from the cells and they were treated with either 100 nM lapatinib (A and D), 250 nM lapatinib (B and E) or 500 nM lapatinib (C and F) and the percentage growth of the cells was compared cells treated with lapatinib only. Growth is expressed relative to control untreated cells. Error bars represent the standard deviation of triplicate experiments. Click here for file

Additional file 4: Figure S3 (A) Proliferation assay in SKBR3-L cells treated with okadaic acid (3 nM) alone and in combination with trastuzumab and (B) cell cycle assay in SKRB3-L treated with okadaic acid (3 nM) with/without lapatinib. Error bars represent the standard deviation of triplicate experiments. Click here for file

Additional file 5: Figure S4 SKBR3 parental cells were pretreated with 2.5 μM FTY720 for 24 hours prior to a 5 day treatment with a range of lapatinib concentrations of lapatinib (0-612.5 nM). Cell growth was measured after 5 days of lapatinib treatment. Growth is expressed relative to control untreated cells. Error bars represent the standard deviation of triplicate experiments. Click here for file
