# Correction: PP2A inhibition overcomes acquired resistance to HER2 targeted therapy

**DOI:** 10.1186/s12943-023-01890-z

**Published:** 2023-10-30

**Authors:** Martina S. J. McDermott, Brigid C. Browne, Neil T. Conlon, Neil A. O’Brien, Dennis J. Slamon, Michael Henry, Paula Meleady, Martin Clynes, Paul Dowling, John Crown, Norma O’Donovan

**Affiliations:** 1 Molecular Therapeutics for Cancer Ireland, National Institute for Cellular Biotechnology, Dublin City University, Glasnevin, Dublin 9 Ireland; 2 Cancer Research Program, The Kinghorn Cancer Centre, Garvan Institute of Medical Research Sydney, Sydney, NSW Australia; 3 Division of Hematology-Oncology, Department of Medicine, David Geffen School of Medicine, University of California at Los Angeles, Los Angeles, CA USA; 4 Department of Biology, National University of Ireland, Maynooth, Maynooth, Co, Kildare Ireland; 5 Department of Medical Oncology, St Vincent’s University Hospital, Elm Park, Dublin 4, Ireland

**Correction: Mol Cancer 13, 157 (2014)**

**https://doi.org/10.1186/1476-4598-13-157**

Following publication of the original article [1], a reader reported that “two signals in Fig. 3C seem to be unexpectedly similar”, and as original blots are no longer available (only cropped versions of the triplicate blots), an alternative replicate of the Fig. 3c blot is provided in this correction. This replacement does not alter the results presented in the text. The correct figure is given below.

**Fig. 3:**
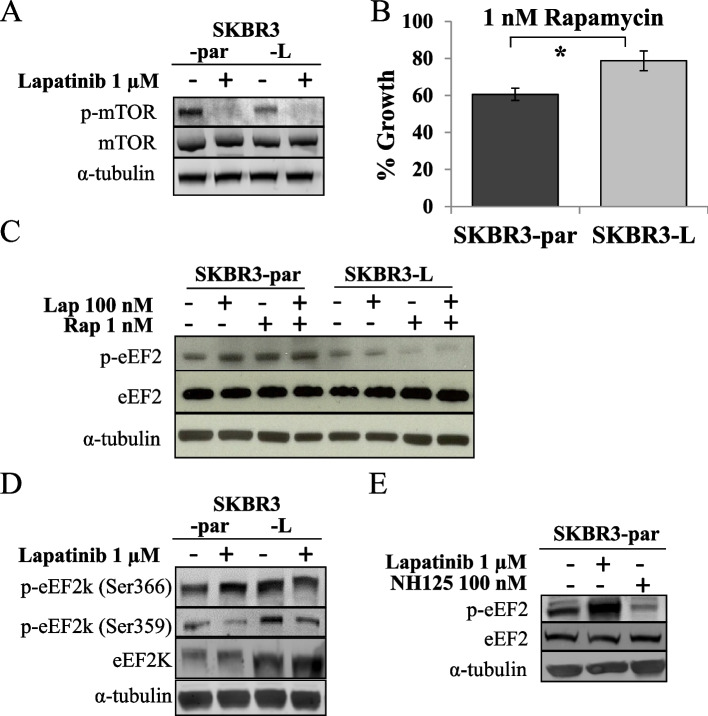
mTOR and eEF2k mediated regulation of eEF2 phosphorylation. **A** Immunoblot analysis of total and phosphorylated mTOR^(Ser2448)^ in SKBR3-par and SKBR3-L cells following 24 h. lapatinib treatment. **B** Effect of rapamycin on growth of SKBR3-par and SKBR3-L cells. Error bars represent the mean ± SD (*n* = 3). **C** Immunoblot analysis of total and phosphorylated eEF2^(Thr56)^ following 24 h. treatment with lapatinib and/or rapamycin. **D** Immunoblot analysis of total and phosphorylated eEF2k^(Ser366, 359)^ in SKBR3-par and SKBR3-L cells following 24 h. lapatinib treatment. **E** Immunoblot examining the effect of NH125 alone and in combination with lapatinib on the phosphorylation of eEF2^(Thr56)^ in SKBR3-par cells. *denotes *p* ≤ 0.05
