# Supplementary figures and images for: PP2A inhibition overcomes acquired resistance to HER2 targeted therapy
Source: Mol Cancer. 2014 Jun 24;13:157. doi: 10.1186/1476-4598-13-157 (PMC4230643; doi:10.1186/1476-4598-13-157)

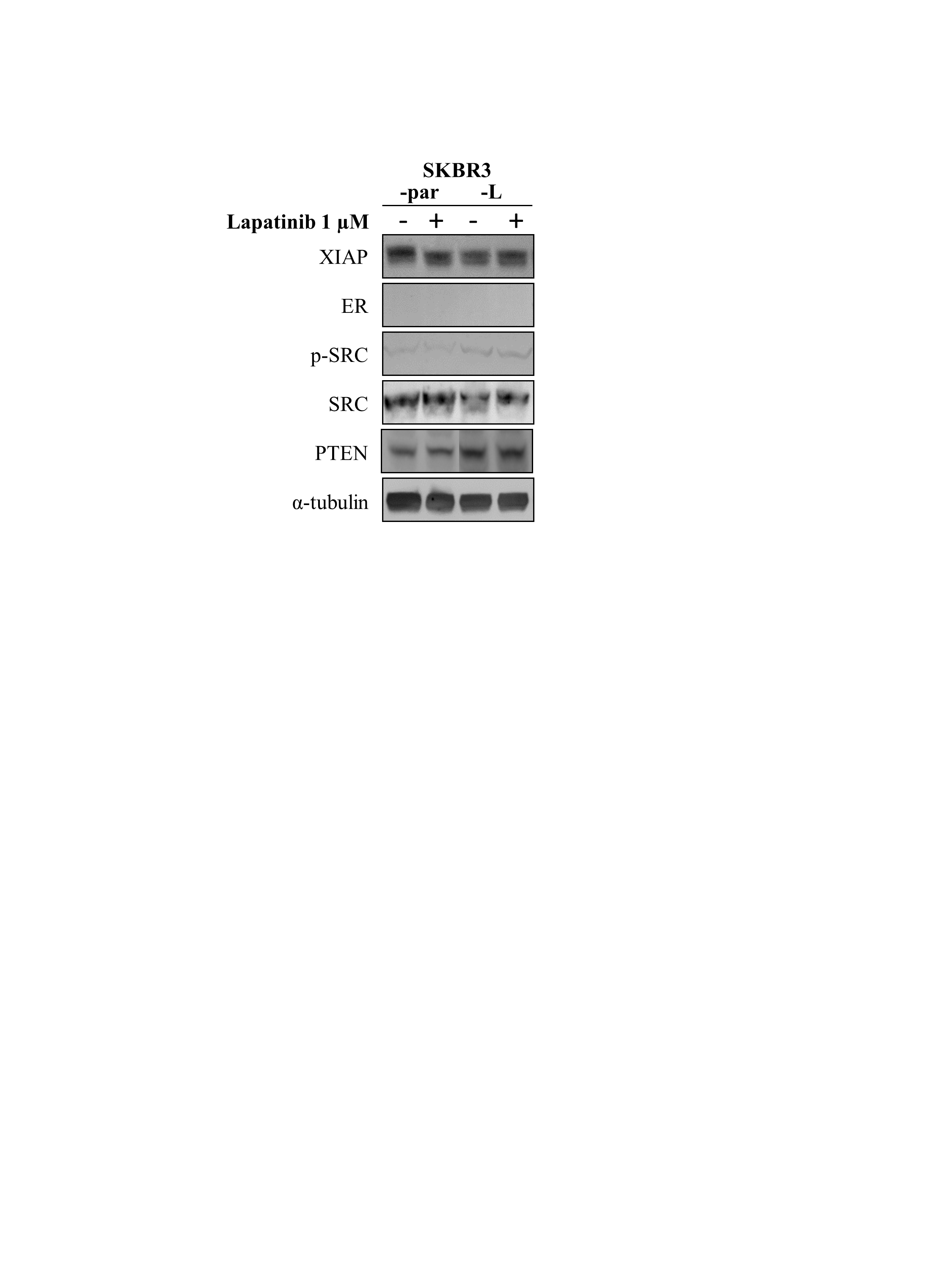

Supplement: Additional file 1: Figure S1 — Analysis of previously published lapatinib resistance mechanisms. Immunoblot analysis of XIAP, estrogen receptor α (ER), total and phosphorylated SRC and PTEN in SKBR3-par and SKBR3-L cells following 24 hr. lapatinib treatment. [file 1476-4598-13-157-S1.tiff]

## Slide 1
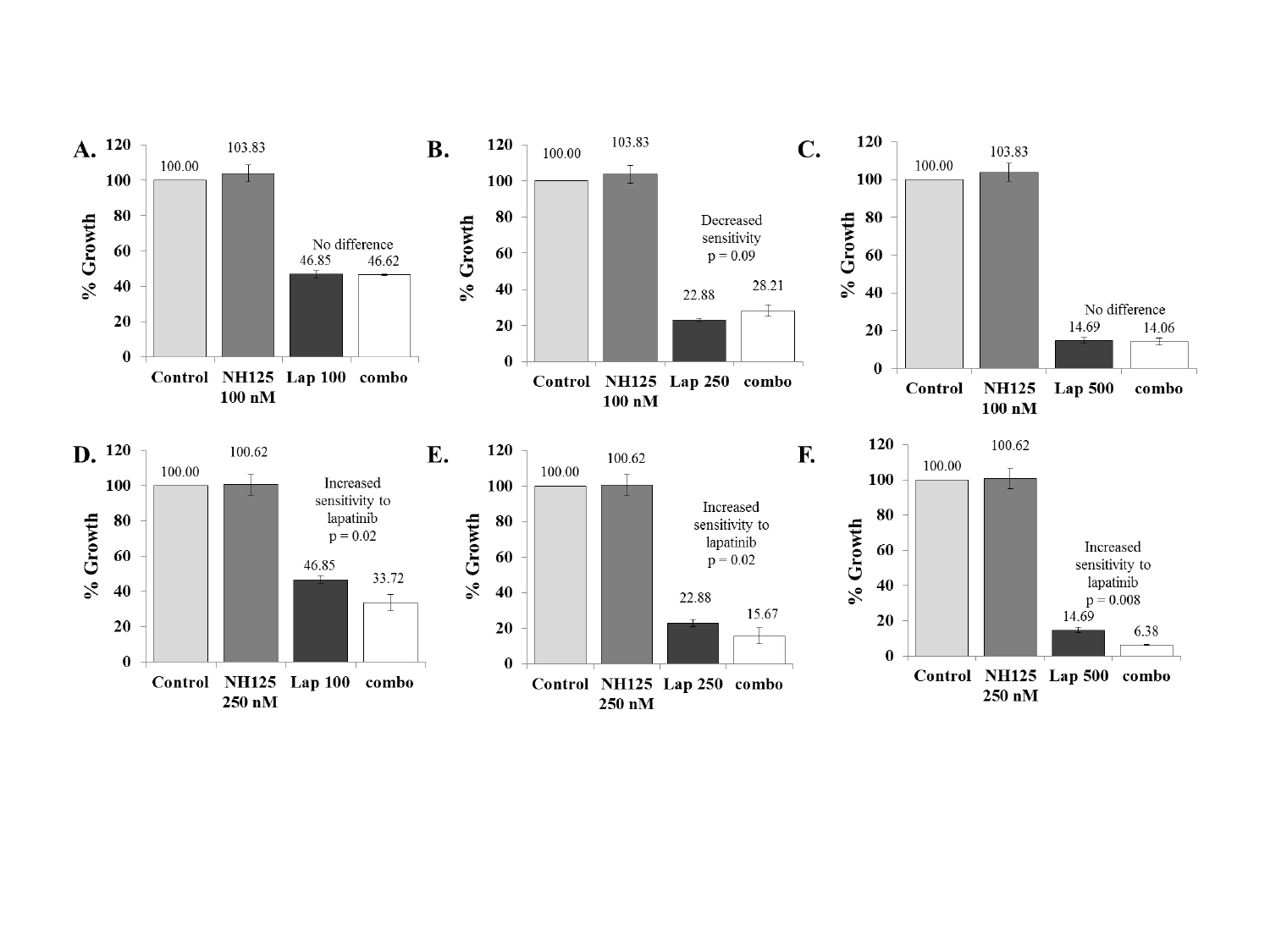

Supplement: Additional file 3: Figure S2 — Concentration dependent effect on NH125 on lapatinib sensitivity. SKBR3-par cells were pre-treated with either 100 nM (A, B and C) or 250 nM (D, E and F) NH125 for 24 hours, after which time NH125 was removed from the cells and they were treated with either 100 nM lapatinib (A and D), 250 nM lapatinib (B and E) or 500 nM lapatinib (C and F) and the percentage growth of the cells was compared cells treated with lapatinib only. Growth is expressed relative to control untreated cells. Error bars represent the standard deviation of triplicate experiments. [file 1476-4598-13-157-S3.pptx]

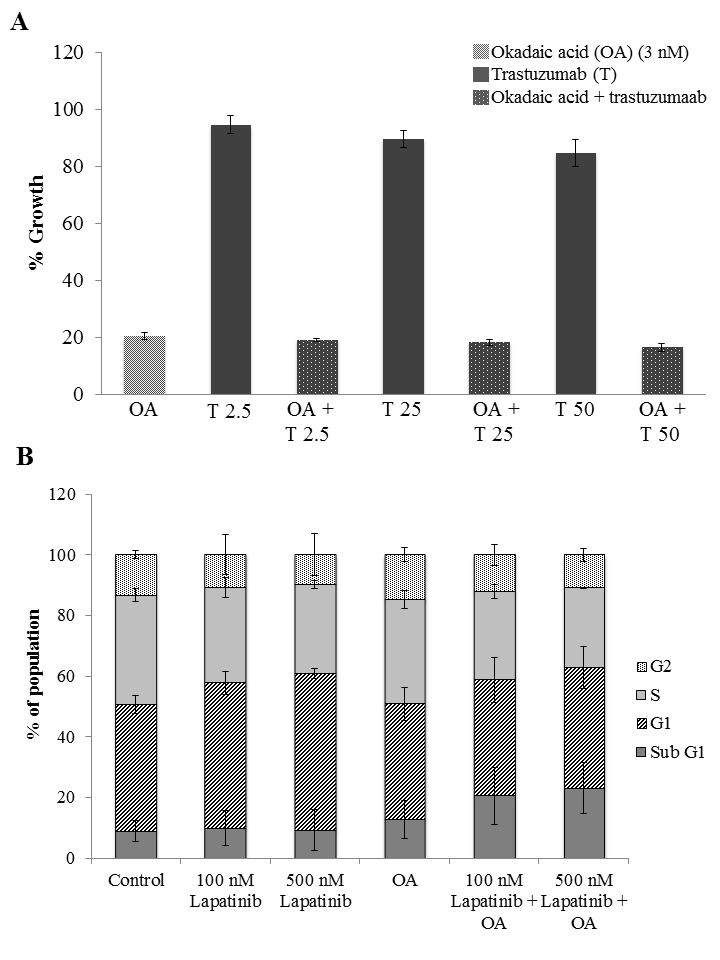

Supplement: Additional file 4: Figure S3 — (A) Proliferation assay in SKBR3-L cells treated with okadaic acid (3 nM) alone and in combination with trastuzumab and (B) cell cycle assay in SKRB3-L treated with okadaic acid (3 nM) with/without lapatinib. Error bars represent the standard deviation of triplicate experiments. [file 1476-4598-13-157-S4.tiff]

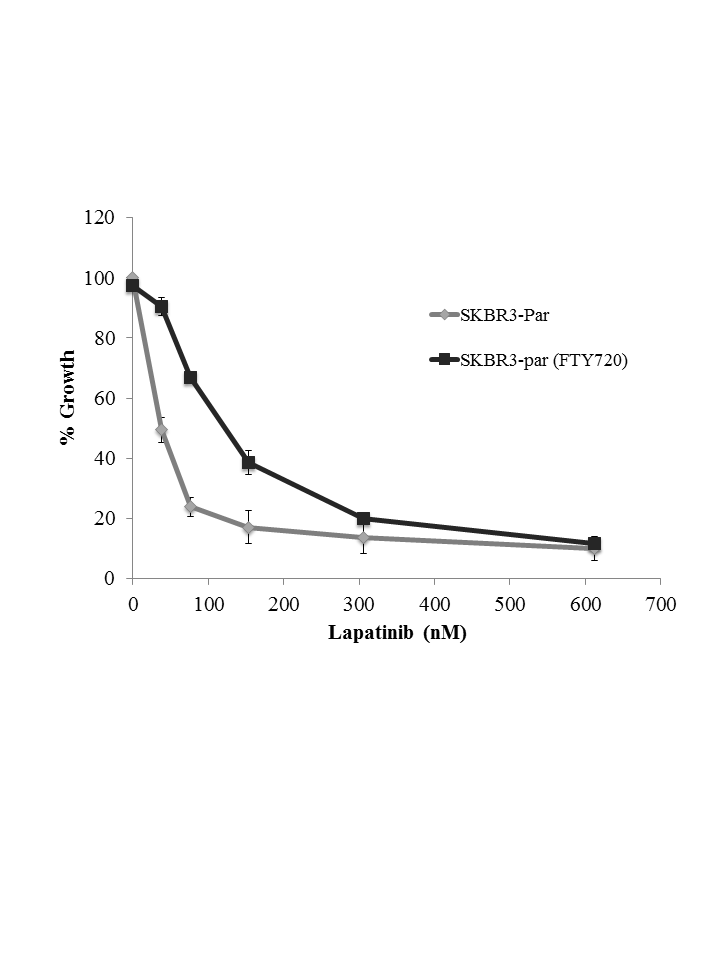

Supplement: Additional file 5: Figure S4 — SKBR3 parental cells were pretreated with 2.5 μM FTY720 for 24 hours prior to a 5 day treatment with a range of lapatinib concentrations of lapatinib (0-612.5 nM). Cell growth was measured after 5 days of lapatinib treatment. Growth is expressed relative to control untreated cells. Error bars represent the standard deviation of triplicate experiments. [file 1476-4598-13-157-S5.tiff]
