# Supplementary material for: PP2A inhibition overcomes acquired resistance to HER2 targeted therapy
Source: Mol Cancer. 2014 Jun 24;13:157. doi: 10.1186/1476-4598-13-157 (PMC4230643; doi:10.1186/1476-4598-13-157)
Supplement: Additional file 2: Table S1 — Phosphoproteins in SKBR3-L compared to SKBR3-par cells. List of identified phosphoproteins with ≥ 1.2-fold increase or decrease in levels in SKBR3-L compared to SKBR3 cells. [file 1476-4598-13-157-S2.docx]

Additional file 2: Table S1: List of identified phosphoproteins with ≥ 1.2-fold increase or decrease in levels in SKBR3-L compared to SKBR3 cells. Fold change: protein abundance in SKBR3-L compared to SKBR3. p-values were calculated using Students’ t-tests, ID numbers refer to MASCOT IDs. The biological processes associated with each of the proteins identified were determined using Panther (<http://www.pantherdb.org/>) [49,50].

| **Spot ID** | **Gene name** | **Ensembl id** | **Protein ID** | **Fold Change** | **t-test p value** | **Biological Process** |
| --- | --- | --- | --- | --- | --- | --- |
| 1945 | CRYAB | ENSG00000263007 | Crystallin; alpha B | 11.26 | 0.010 | Immune system process; protein folding; muscle contraction; visual perception; sensory perception; response to stress |
| 1921 | CRYAB | ENSG00000263007 | Crystallin; alpha B | 7.74 | 0.002 | Immune system process; protein folding; muscle contraction; visual perception; sensory perception; response to stress |
| 2203 | S100A8 | ENSG00000143546 | S100 calcium binding protein A8 (calgranulin A) | 3.97 | 0.009 | Macrophage activation; DNA replication; cell cycle; cell communication |
| 2166 | S100A9 | ENSG00000163220 | Chain H; Crystal structure of Mrp14 complexed with chaps | 3.67 | 0.005 | Macrophage activation; DNA replication; cell cycle; cell communication |
| 2152 | S100A9 | ENSG00000163220 | Chain H; Crystal structure of Mrp14 complexed with chaps | 3.61 | 0.015 | Macrophage activation; DNA replication; cell cycle; cell communication |
| 2213 | S100A8 | ENSG00000143546 | S100 calcium binding protein A8 (calgranulin A) | 3.46 | 0.009 | Macrophage activation; DNA replication; cell cycle; cell communication |
| 2145 | S100A9 | ENSG00000163220 | Chain H; Crystal structure of Mrp14 complexed with chaps | 3.24 | 0.007 | Macrophage activation; DNA replication; cell cycle; cell communication |
| 580 | SCIN | ENSG00000006747 | Adseverin | 2.57 | 0.012 | Cellular process; cellular component morphogenesis; cellular component organization |
| 1797 | HSPBAP1 | ENSG00000169087 | Heat shock 27kDa protein 1 | 2.38 | 0.037 | Cell growth; cell differentiation |
| 461 | HSP90B1 | ENSG00000166598 | Tumor rejection antigen (gp96) 1 | 2.16 | 0.005 | Protein folding; response to stress |
| **Spot ID** | **Gene name** | **Ensembl id** | **Protein ID** | **Fold Change** | **t-test p value** | **Biological Process** |
| 366 | HSP90B1 | ENSG00000166598 | Tumor rejection antigen (gp96) 1 | 1.92 | 0.016 | Protein folding; response to stress |
| 1789 | HMGB1 | ENSG00000189403 | High-mobility group box 1 | 1.67 | 0.014 | Transcription from RNA polymerase II promoter; cell communication; regulation of transcription from RNA polymerase II promoter; chromatin organization |
| 925 | FKBP4 | ENSG00000004478 | FK506 binding protein 4 | 1.6 | 0.006 | Cellular protein modification process; cellular process |
| 1467 | UNG | ENSG00000076248 | Uracil DNA glycosylase | 1.52 | 0.014 | Metabolic process; response to stress |
| 1462 | UNG | ENSG00000076248 | Uracil DNA glycosylase | 1.46 | 0.003 | Metabolic process; response to stress |
| 951 | FKBP4 | ENSG00000004478 | FK506 binding protein 4 | 1.45 | 0.018 | Cellular protein modification process; cellular process |
| 1647 | LACTB2 | ENSG00000147592 | Lactamase; beta 2 | 1.44 | 0.014 | Metabolic process |
| 1486 | GAPDH | ENSG00000111640 | Glyceraldehyde-3-phosphate dehydrogenase | 1.35 | 0.032 | Glycolysis |
| 1688 | MTAP | ENSG00000099810 | Methylthioadenosine phosphorylase | 1.33 | 0.014 | Purine nucleobase metabolic process |
| 369 | EEF2 | ENSG00000167658 | Eukaryotic translation elongation factor 2 | 1.22 | 0.040 | Translation; Regulation of translation |
| 1720 | PSMA4 | ENSG00000041357 | PSMA4 protein | -1.21 | 0.015 | Proteolysis |
| 1750 | PSMA3 | ENSG00000100567 | Proteasome (prosome; macropain) subunit; alpha type; 3 | -1.24 | 0.006 | Proteolysis |
| 1909 | PSMB6 | ENSG00000142507 | Proteasome subunit Y | -1.26 | 0.000 | Proteolysis |
| 1774 | PSMA7 | ENSG00000101182 | Proteasome (prosome; macropain) subunit; alpha type; 7 | -1.28 | 0.003 | Proteolysis |
| 844 | STIP1 | ENSG00000168439 | Stress-induced-phosphoprotein 1(Hsp70/90-organizing protein) | -1.31 | 0.040 | Protein folding; response to stress |
| **Spot ID** | **Gene name** | **Ensembl id** | **Protein ID** | **Fold Change** | **t-test p value** | **Biological Process** |
| 1816 | PSMB4 | ENSG00000159377 | Proteasome (prosome; macropain) subunit; beta type; 4 | -1.34 | 0.043 | Proteolysis |
| 1135 | PSMC2 | ENSG00000161057 | Proteasome (prosome; macropain) 26S subunit; ATPase; 2 | -1.34 | 0.004 | Proteolysis |
| 783 | HSPA1A | ENSG00000231555 | HSPA1A protein; HSPA1A protein | -1.36 | 0.012 | Immune system process; protein folding; protein complex assembly; response to stress; protein complex biogenesis |
| 1701 | PSMB7 | ENSG00000136930 | PSMB7 protein | -1.37 | 0.016 | Proteolysis |
| 982 | G6PD | ENSG00000160211 | Glucose-6-phosphate dehydrogenase | -1.41 | 0.034 | Monosaccharide metabolic process |
| 361 | EEF2 | ENSG00000167658 | Eukaryotic translation elongation factor 2 | -1.46 | 0.005 | Translation; Regulation of translation |
| 1484 | OSGEPL1 | ENSG00000128694 | Putative sialoglycoprotease | -1.48 | 0.015 | Proteolysis |
| 985 | G6PD | ENSG00000160211 | Glucose-6-phosphate dehydrogenase | -1.64 | 0.020 | Monosaccharide metabolic process |
| 338 | EEF2 | ENSG00000167658 | Eukaryotic translation elongation factor 2 | -1.68 | 0.001 | Translation; Regulation of translation |
| 354 | EEF2 | ENSG00000167658 | Eukaryotic translation elongation factor 2 | -1.68 | 0.000 | Translation; Regulation of translation |
| 1006 | G6PD | ENSG00000160211 | Glucose-6-phosphate dehydrogenase | -1.72 | 0.010 | Monosaccharide metabolic process |
| 948 | CAP1 | ENSG00000131236 | Adenylyl cyclase-associated protein | -1.98 | 0.003 | Cell communication |
| 1659 | NQO1 | ENSG00000181019 | Chain D; Crystal structure of human Dt-Diaphorase | -1.99 | 0.026 | Metabolic process |
| 340 | EEF2 | ENSG00000167658 | Eukaryotic translation elongation factor 2 | -1.99 | 0.000 | Translation; Regulation of translation |
| **Spot ID** | **Gene name** | **Ensembl id** | **Protein ID** | **Fold Change** | **t-test p value** | **Biological Process** |
| 341 | EEF2 | ENSG00000167658 | Eukaryotic translation elongation factor 2 | -2 | 0.002 | Translation; Regulation of translation |
| 350 | EEF2 | ENSG00000167658 | Eukaryotic translation elongation factor 2 | -2.42 | 0.000 | Translation; Regulation of translation |
